# A Digital Patient Engagement and Monitoring System to Improve Quality, Safety, and Continuity of Care for Pediatric Sickle Cell Anemia: A Cross-Sectional Study of Caregiver Perceptions

**DOI:** 10.7759/cureus.101590

**Published:** 2026-01-15

**Authors:** Ehab Hanafy, Badriah G Alasmari, Abdelhakam A Elmugadam, Sara S Hassanien, Ayman S Alsafy, Mohammed Alpakra

**Affiliations:** 1 Oncology and Hematology, Armed Forces Hospital Southern Region, Khamis Mushayt, SAU; 2 Pediatrics, Armed Forces Hospital Southern Region, Khamis Mushayt, SAU

**Keywords:** care continuity, digital health, medication adherence, mhealth, patient engagement, pediatrics, remote monitoring, sickle-cell anemia

## Abstract

Introduction

The management of chronic conditions like sickle cell anemia (SCA) requires high levels of patient and caregiver engagement to ensure adherence to treatment and prevent complications. Digital health interventions offer a promising avenue to support this engagement. This study evaluates the awareness, perceived usefulness, and readiness of caregivers to adopt a digital patient engagement and monitoring system for pediatric SCA management.

Methods

A cross-sectional survey was conducted using a structured Arabic-language questionnaire administered to 165 primary caregivers of children with SCA. The instrument included closed-ended Likert-scale items on current care challenges and digital readiness, alongside open-ended questions for qualitative insights. Quantitative data were analyzed descriptively with 95% confidence intervals, and qualitative responses underwent thematic analysis.

Results

Participants were predominantly parents (95.7%) and well educated (84.2% secondary or university graduates). Although 74.5% reported that clinic information was sufficient, adherence challenges remained: 44.2% sometimes and 11.5% frequently forgot appointments or medications, while 41.2% reported at least one missed event within six months. Perceptions toward digital tools were highly favourable: 86.1% (95% CI 80.2-90.5) endorsed their usefulness, 92.1% (95% CI 87.0-95.4) expressed readiness to use them, and 89.7% (95% CI 84.6-93.8) trusted digital platforms to manage health data. Nearly all respondents (97%) welcomed mobile notifications. Thematic analysis (n = 105 comments) identified four dominant needs: automated reminders (31%), emergency guidance (28%), improved communication (22%), and access to laboratory results (24%).

Conclusion

Caregivers of children with SCA demonstrate high readiness and a strong positive perception toward a digital engagement system. Such a system is well-positioned to address critical gaps in adherence and communication, thereby potentially improving the quality, safety, and continuity of care. The alignment between desired features and the system's design suggests high potential for successful implementation and impact.

## Introduction

Sickle cell anemia (SCA) is a chronic, inherited hemoglobinopathy characterized by recurrent vaso-occlusive pain crises, progressive end-organ damage, and significant morbidity and mortality throughout the patient’s lifespan [1]. Pediatric SCA management is especially complex, demanding meticulous adherence to prophylactic medications such as penicillin, scheduled immunizations, hydroxyurea therapy, and regular follow-up for screening and early intervention against complications, including stroke, infection, and organ dysfunction [2]. These continuous care demands impose a considerable burden on families, particularly caregivers who serve as the primary coordinators of daily treatment adherence and health maintenance.

Despite standardized protocols and multidisciplinary follow-up clinics, gaps in the continuity of care remain a major concern. Missed appointments, delayed medication refills, and incomplete prophylaxis have been linked to higher rates of emergency visits, hospitalizations, and preventable morbidity [3]. Non-adherence in SCA has multifactorial origins, ranging from forgetfulness, socioeconomic barriers, and caregiver fatigue to fragmented communication with healthcare providers [4]. Traditional outpatient models, while foundational to care delivery, are inherently episodic and limited in providing the continuous, real-time support needed between clinic visits.

In recent years, digital transformation in healthcare has introduced a paradigm shift in how chronic conditions are managed. Digital Patient Engagement and Monitoring Systems (DPEMS), an umbrella term encompassing mobile health (mHealth) applications, telemonitoring platforms, and automated communication tools, enable continuous connectivity between patients, caregivers, and providers [5]. These technologies have demonstrated potential to enhance education, reinforce adherence, deliver reminders, and enable timely interventions, thereby transforming chronic care from a reactive to a proactive model [6]. In particular, mHealth interventions leverage smartphones and wearable sensors to deliver real-time data and personalized feedback, improving both self-management and clinical outcomes [7, 8].

Evidence from diverse chronic conditions supports this approach. In diabetes and asthma, mobile reminders and educational modules have improved medication adherence, glycemic control, and patient empowerment [7, 8]. Similarly, interventions in hypertension management have demonstrated reductions in systolic blood pressure and improved follow-up attendance [9]. In pediatric SCA, although the research base is smaller, early studies indicate clear benefits: Short Message Service (SMS)-based reminders have significantly improved appointment attendance [10], while web-based educational tools have enhanced disease knowledge, caregiver confidence, and self-efficacy [11].

The primary advantage of these systems lies in their ability to deliver just-in-time support, providing information or prompts exactly when needed to prevent crises and reinforce adherence. For conditions like SCA, where acute exacerbations can arise unpredictably, such timely digital interactions can bridge the gap between clinic visits and daily home management.

Nonetheless, substantial challenges accompany the deployment of digital health tools. The digital divide - disparities in access to technology or digital literacy - can exacerbate health inequities, especially in low-resource or rural populations [12]. In addition, data privacy, information security, and usability remain recurrent concerns. Poorly designed user interfaces or burdensome data entry can reduce engagement and contribute to high dropout rates [13]. On a systems level, many pilot mHealth initiatives fail to achieve scale because of weak integration with electronic health records (EHRs), lack of clinician buy-in, and insufficient long-term funding models [14, 15].

Given these challenges, the success of any digital health solution depends critically on end-user acceptance and readiness. Understanding the perceptions, needs, and expectations of caregivers who are both facilitators and gatekeepers of pediatric care is essential before implementation [16].

Accordingly, this study was designed to evaluate the awareness, perceived usefulness, trust, and readiness of caregivers of pediatric SCA patients to adopt a structured Digital Patient Engagement and Monitoring System.

By integrating both quantitative and qualitative insights, the study seeks to identify key barriers, facilitators, and feature priorities, thereby informing the design of future digital interventions that enhance adherence, communication, and continuity of care for children living with SCA.

## Materials and methods

Study design and setting

A descriptive cross-sectional study was conducted to assess caregivers’ awareness, perceptions, and readiness to adopt a digital follow-up and education system for patients with sickle cell anemia (SCA).

The study took place at the Armed Forces Hospital Southern Region (AFHSR) in Khamis Mushayt, Saudi Arabia, which provides comprehensive pediatric and adolescent hematology services, including a specialized Sickle Cell Clinic staffed by hematologists, pediatricians, nurses, and patient educators. The clinic offers regular multidisciplinary follow-up, medication monitoring, and family counseling for patients with confirmed SCA across the southern region.

Study population and sampling

The study population comprised parents and primary caregivers of patients with confirmed sickle cell anemia (HbSS) who were enrolled in the AFHSR Sickle Cell Clinic and actively followed during the study period.

The inclusion criteria comprised caregivers (parents, legal guardians, or close family members) of patients diagnosed with SCA and registered in the AFHSR hematology database, caregivers whose patients had regular follow-up in the Sickle Cell Clinic for at least six months prior to data collection, and caregivers who were able to read and understand Arabic and agreed to participate voluntarily. The exclusion criteria included caregivers of patients with other hemoglobinopathies, such as hemoglobin SC disease or β-thalassemia, as well as families who were not yet enrolled in the clinic’s follow-up program or who submitted incomplete or duplicate responses. A total of 165 fully completed questionnaires met the inclusion criteria and were included in the final analysis.

Data collection instrument

A structured Arabic-language questionnaire was developed by the investigator after reviewing previous literature on mHealth adoption and adherence in chronic diseases [7, 8, 11]. 

Content and face validity were established through independent review by two consultant hematologists, who assessed the questionnaire for clinical relevance, clarity, and appropriateness for the target population. Minor revisions were made based on their feedback prior to finalization.

The tool was pre-tested with a small sample of caregivers (n = 10) to ensure linguistic clarity and cultural appropriateness before formal administration.

Reliability analysis was conducted for the Likert-scale items that measure a single construct related to perceived usefulness, ease of use, trust, and acceptance of the electronic health application. No questionnaire items were deleted. Single-item questions and items assessing different constructs were analytically excluded from the internal consistency analysis. The Likert agreement subscale demonstrated excellent internal consistency (Cronbach’s α = 0.964, pilot sample n = 10).

The questionnaire was intended for descriptive research purposes and did not involve diagnostic or interventional assessment.

The final instrument comprised three sections, including demographic characteristics covering patient gender, age, duration of follow-up at the clinic, and caregiver educational level; current care challenges addressing satisfaction with the adequacy of available clinical information, the frequency of difficulties remembering appointments or medications, and the number of missed appointments or medication doses during the preceding six months; and perceptions of the digital system, which were assessed using five-point Likert scales ranging from strongly disagree (1) to strongly agree (5) to evaluate perceived usefulness, trust, expected impact on care, and readiness to adopt a digital system, in addition to open-ended questions that captured qualitative insights regarding preferred features and potential concerns. Questionnaires were distributed to caregivers, and participation was voluntary, anonymous, and had no impact on clinical care.

Ethical considerations

Ethical approval was obtained from the Institutional Review Board, Armed Forces Health Services (approval number H-06-KM-001). The study followed the principles of the Declaration of Helsinki (2013 revision) and local institutional research-governance guidelines.

A waiver of informed consent was granted, as the study involved no collection or disclosure of personally identifiable patient information and posed no risk to participant privacy or confidentiality.

Data analysis

Quantitative data were analyzed using IBM SPSS Statistics version 26 (IBM Corp., Armonk, NY, USA). Descriptive statistics were expressed as frequencies, percentages, and 95% confidence intervals. Responses of Strongly Agree and Agree were combined to represent positive perception, while Disagree and Strongly Disagree were grouped as negative perception.

Exploratory analyses examined associations between caregiver characteristics (education level, follow-up duration) and major outcomes (readiness, trust, and missed events) using Pearson’s χ² test; significance was set at p < 0.05 for exploratory interpretation only.

Qualitative data from open-ended responses were analyzed using thematic content analysis. Two reviewers independently coded responses to identify recurring ideas, which were then consolidated into major themes representing caregiver priorities and expectations. Consensus validation ensured coding reliability.

## Results

Demographic characteristics

A total of 165 caregivers of children with sickle-cell anemia participated in the survey. Among the children, 73 (44.2%) were male and 92 (55.8%) female. Most children were in the younger age brackets, with 79 (47.9%) aged 1-5 years, 53 (32.1%) aged 6-10 years, and 20 (12.1%) aged 11-14 years, while a small proportion (13 children, 7.9%) were older than 14 years.

The majority of respondents (157 caregivers, 95.7%) were parents or legal guardians, ensuring that the responses reflected the perceptions of those directly responsible for daily care.

Duration of clinical follow-up was evenly distributed: 48 (29.1%) families had been followed for less than one year, 50 (30.3%) for one to three years, and 67 (40.6%) for more than three years.

Educational attainment was generally high, with 69 (41.8%) caregivers completing secondary school and 70 (42.4%) holding university degrees. Only 19 (11.5%) had a primary school education, and seven (4.2%) had intermediate education. These findings confirm that the surveyed caregivers were predominantly literate and technologically capable, forming a suitable target population for future digital health initiatives.

Current care challenges and adherence gaps

When asked whether current clinical follow-up provides sufficient information about the child’s condition, 123 (74.5%; 95% CI 67.4-80.5) stated that it was “completely sufficient,” while 41 (24.8%) considered it “to some extent.”

Despite this high satisfaction, 92 caregivers (55.7%; 95% CI 48.0-63.1) reported sometimes or often struggling to remember medications or appointments - 73 (44.2%) occasionally and 19 (11.5%) frequently.

During the preceding six months, 68 caregivers (41.2%; 95% CI 33.9-49.0) reported missing at least one medication dose or appointment, most often one to three times, yet this pattern suggests that nearly half of the families experience occasional adherence gaps that could be mitigated by structured reminder systems (Table 1).

**Table 1:** Overall caregiver responses (n = 165) n (%) = number and percentage of respondents; 95% CI = 95% confidence interval; agree/strongly agree = combined positive Likert responses; ≥ = greater than or equal to.

Measure	n (%) positive	95% CI (%)
Perceived usefulness (agree / strongly agree)	142 (86.1)	80.2-90.5
Trust in digital tools (agree / strongly agree)	148 (89.7)	84.6-93.8
Readiness to adopt (agree / strongly agree)	152 (92.1)	87.0-95.4
Missed ≥ 1 appointment or dose in past 6 months	68 (41.2)	33.9-49.0

Qualitative comments attributed lapses mainly to divided caregiving responsibilities, competing work duties, or lack of coordinated reminder tools, underscoring the need for reliable, family-wide adherence supports.

Perceptions of digital health tools

Perceived Usefulness

When asked whether a digital health tool would help them remember medications and appointments, 58 (35.1%) strongly agreed, and 84 (50.9%) agreed, while 12 (7.2%) were neutral and 11 (6.6%) disagreed or strongly disagreed. Overall, 142 caregivers (86.1%; 95% CI 80.2-90.5) endorsed the usefulness of digital systems for improving adherence and follow-up consistency.

Readiness for Use

A high level of willingness to adopt digital tools was recorded: 83 (50.3%) strongly agreed, and 69 (41.8%) agreed, producing a readiness index of 92.1% (95% CI 87.0-95.4). Only two caregivers (1.2%) disagreed, and 11 (6.7%) were neutral. Readiness remained consistently high across all age and follow-up categories.

Trust and Information Security

Confidence in digital recordkeeping was likewise strong. For the statement “I trust a digital platform to handle health information securely,” 59 (35.7%) strongly agreed, and 89 (53.9%) agreed, giving a trust index of 89.7% (95% CI 84.6 - 93.8). Only 17 caregivers (10.3%) were neutral or disagreed, confirming broad comfort with the confidentiality of digital data management.

Expected Impact on Care

Perceived clinical benefit was equally robust. In response to “Using a digital system would help reduce unnecessary hospital visits,” 147 (88.9%; 95% CI 83.2-92.8) agreed or strongly agreed, showing clear anticipation of improved care coordination and resource efficiency.

Notification Preferences

Nearly all caregivers were receptive to phone-based notifications: 160 (97%; 95% CI 93.1-98.9) welcomed receiving reminders or alerts, three (1.8%) were uncertain, and two (1.2%) declined. Respondents emphasized the importance of timely and bilingual notifications (Arabic and English) to accommodate diverse family preferences.

Stratified findings

To explore whether caregiver perceptions differed by demographic or clinical background, proportions were stratified by the child's age group and the duration of follow-up (Table 2).

**Table 2:** Stratified proportions (%) with 95% confidence interval Values are percentages with 95% confidence intervals in parentheses. n = number of participants. + indicates a positive response (agree/strongly agree). ≥ = greater than or equal to. Missed ≥ 1 indicates at least one missed appointment or medication dose during the specified period.

	Usefulness +	Trust +	Readiness +	Missed ≥ 1
Age (years)				
1-5 (n = 79)	85.7 (76.3-91.9)	86.9 (77.8-92.7)	93.4 (85.7-97.3)	39.2 (28.6-50.9)
6-10 (n = 53)	88.7 (76.7-95.0)	86.8 (74.4-93.7)	90.6 (79.2-96.2)	43.4 (30.4-57.4)
11-14 (n = 20)	85.0 (62.1-95.0)	85.0 (62.1-95.0)	90.0 (68.3-97.6)	40.0 (20.3-63.6)
>14 (n = 13)	84.6 (57.8-95.7)	84.6 (57.8-95.7)	92.3 (66.7-98.6)	38.5 (17.7-64.5)
Follow-up				
<1 year (n = 48)	83.3 (70.2-91.3)	85.4 (72.4-92.8)	89.6 (77.6-95.7)	43.8 (30.2-58.4)
1-3 years (n = 50)	88.0 (75.9-94.6)	88.0 (75.9-94.6)	94.0 (83.5-97.9)	42.0 (28.9-56.4)
>3 years (n = 67)	86.6 (76.4-92.8)	88.1 (78.4-93.9)	92.5 (83.6-96.8)	38.8 (27.9-50.8)

Across age groups, perceived usefulness ranged from 84.6% to 88.7%, trust from 84.6% to 86.9%, and readiness from 90.0% to 93.4%. Adherence lapses (missed ≥ 1 event) were similar across ages (approximately 39-43%), with overlapping 95% CIs, indicating no meaningful differences.

Across follow-up duration, usefulness ranged from 83-88%, trust from 85-88%, and readiness from 90-94%. Caregivers followed for >3 years continued to express very high readiness (92.5%) and trust (88.1%), suggesting that sustained clinic contact does not diminish enthusiasm for digital solutions.

Exploratory associations

To further assess potential predictors of caregiver responses, χ² tests examined the relationship between education level and follow-up duration with readiness, trust, and missed ≥ 1 event (Table 3).

**Table 3:** Exploratory χ² tests (education and follow-up vs key outcomes) χ² = chi-square test statistic; df = degrees of freedom; p = p-value. + indicates a positive outcome (agree/strongly agree). Missed ≥ 1 indicates at least one missed appointment or medication dose. Not significant denotes p ≥ 0.05; trend toward association indicates borderline statistical significance.

Predictor	Outcome	χ² (df)	p	Interpretation
Education	Readiness +	2.54 (3)	0.468	No association
Education	Trust +	1.43 (3)	0.698	No association
Education	Missed ≥ 1	5.65 (3)	0.130	Not significant
Follow-up	Readiness +	4.81 (2)	0.091	Trend toward association (p ≈ 0.09)
Follow-up	Trust +	2.74 (2)	0.254	No association
Follow-up	Missed ≥ 1	0.35 (2)	0.840	No association

No statistically significant associations were observed between education and readiness (χ² = 2.54, df = 3, p = 0.47), trust (χ² = 1.43, p = 0.70), or missed ≥ 1 event (χ² = 5.65, p = 0.13). Similarly, follow-up duration showed no significant relationship with trust (χ² = 2.74, p = 0.25) or missed ≥ 1 event (χ² = 0.35, p = 0.84).

A borderline trend was noted for follow-up vs readiness (χ² = 4.81, df = 2, p = 0.09), with slightly higher readiness among those followed longer, but confidence intervals overlapped substantially, indicating only a weak, non-significant tendency.

Qualitative findings

**Figure 1:**
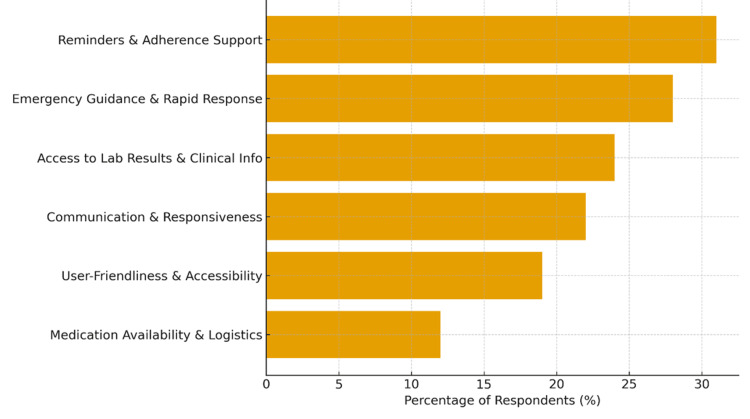
Thematic Map of Caregiver Priorities Bars represent the percentage of respondents (%) selecting each priority domain. Percentages reflect the proportion of participants endorsing the item.

A total of 105 caregivers (63.6% of all respondents) provided open-ended comments describing their needs and expectations from a digital follow-up system. Responses were analyzed thematically after translation from Arabic into English. Four core themes and two supporting subthemes emerged, reflecting practical and emotional dimensions of caregiving (Figure 1).

Theme 1: Reminders and Adherence Support (Reported by ≈31%)

The most dominant theme involved the need for automated reminders for medications and clinic appointments. Caregivers emphasized that consistent alerts would help avoid missed visits and doses, particularly in households where both parents share responsibilities.

“A reminder for appointments and medicines would help us stay organized, especially when both parents are busy.”

“It should notify both parents - if one forgets, the other remembers.”

Several participants also requested dose-specific alerts and distinguishable sound or color signals for urgent reminders. This reflects a strong preference for structured, bilingual, and family-linked reminder systems.

Theme 2: Emergency Guidance and Rapid Response (≈28%)

Many caregivers expressed concern about what to do during acute pain crises or fever episodes. They desired a function within the app to provide step-by-step instructions and to clarify when to seek immediate hospital care.

“I want it to tell me quickly if the child’s situation needs hospital attention or not.”

“Sometimes we panic; the app should explain dangerous symptoms and the right action.”

This theme highlights the anxiety surrounding unpredictable complications in SCA and the need for instant, evidence-based crisis guidance.

Theme 3: Access to Laboratory Results and Clinical Information (≈24%)

Caregivers frequently requested easy access to laboratory results, transfusion records, and growth or medication summaries. They viewed such access as crucial for understanding progress and communicating effectively with clinicians.

“I want to see the child’s lab results and know if things are improving.”

“It should show test results and doctor notes in simple language.”

This underscores a demand for transparency and educational visualization of clinical data to promote shared decision-making.

Theme 4: Communication and Responsiveness (≈22%)

Participants emphasized the importance of direct communication with the medical team, preferably through a secure messaging or consultation feature. Delayed responses from hospital staff were a recurring frustration.

“When I send a question, I hope someone replies quickly.”

“Contacting the doctor for advice should be easier.”

This indicates the desire for a two-way interactive channel that maintains continuity and reduces unnecessary visits through timely remote reassurance.

Subtheme A: Medication Availability and Logistics

A subset of caregivers mentioned issues beyond digital communication, particularly medication refill delays, notably hydroxyurea, and requested that the system link with the hospital pharmacy to track or request refills.

“Please ensure hydroxyurea is available; remind us when to refill.”

Subtheme B: User-Friendliness, Language, and Accessibility (≈19%)

Respondents stressed that the platform must be simple, ad-free, and cost-free, with clear Arabic and English options. They emphasized inclusivity for less technologically experienced users.

“It should be easy to use and without advertisements.”

“Make it free so all families can benefit.”

This theme reinforces the need for equitable design, intuitive navigation, culturally appropriate language, and minimal technical barriers.

Collectively, caregivers prioritized reminder reliability, emergency guidance, result transparency, and communication responsiveness as the most critical features of a digital SCA management system. These insights mirror global caregiver expectations in chronic disease mHealth studies [9], confirming that any successful implementation must combine educational content, interactive communication, and user-centered accessibility to achieve sustained engagement.

Interpretation

The integrated quantitative and qualitative findings demonstrate a high level of digital readiness and trust among caregivers of children with sickle-cell anemia. Perceived usefulness (86.1%; 95% CI 80.2-90.5), readiness (92.1%; 95% CI 87.0-95.4), and trust (89.7%; 95% CI 84.6-93.8) were all strongly positive (Table 1).

Adherence lapses affected about two in five families, irrespective of education or follow-up duration, suggesting that behavioral rather than demographic factors drive these gaps. The near-universal acceptance of notification-based reminders (97%; 95% CI 93.1-98.9) underscores a clear opportunity for digital engagement.

Overall, the data provide solid evidence of caregiver engagement, a consistent pattern of moderate adherence challenges, and broad endorsement of digital approaches to enhance continuity, safety, and efficiency in pediatric sickle-cell care.

## Discussion

This study demonstrates strong caregiver readiness and high receptivity toward digital engagement tools for pediatric sickle cell anemia (SCA) management. The results reveal not only the feasibility but also the urgency of integrating structured digital systems into chronic care pathways. The convergence between quantitative and qualitative findings underscores that caregiver needs, expectations, and perceptions align closely with global evidence on the transformative potential of digital health technologies.

Strengths and usefulness of the intervention

The study’s findings reaffirm that the principal challenges in pediatric SCA care, namely medication non-adherence and missed appointments, remain major determinants of preventable morbidity, as previously reported by Walsh et al. [3] and Drotar [4]. In this cohort, over one-third of caregivers acknowledged recent lapses in adherence, validating the magnitude of this gap. The near-universal acceptance of reminder notifications (97%) highlights a clear behavioral target that digital patient-engagement and monitoring systems (DPEMS) are ideally positioned to address.

Moreover, caregiver responses demonstrate enthusiasm for a multi-functional digital ecosystem that transcends reminders alone. Qualitative themes revealed a desire for emergency guidance, laboratory result access, and bidirectional communication - functions consistent with evidence that mobile health (mHealth) interventions improve adherence, self-efficacy, and health literacy in chronic diseases [5, 6, 7]. Studies in other pediatric conditions and among SCA adolescents (e.g., iManage) have shown that co-designed mobile applications significantly enhance user engagement and disease self-management [11].

The expectation by nearly 89% of caregivers that such systems could reduce unnecessary hospital visits mirrors previous findings that structured digital follow-up decreases emergency department utilization and unplanned admissions [2, 8]. Collectively, these data suggest that well-implemented digital interventions can optimize adherence, reduce healthcare burden, and strengthen continuity of care - key pillars of quality and patient safety in chronic disease management.

Weaknesses and challenges

Despite the overwhelmingly positive attitudes, several limitations must be acknowledged. The sample was predominantly composed of caregivers with secondary or university education, indicating high digital literacy that may not reflect all populations affected by SCA. This demographic skew introduces a potential selection bias common in digital health research [12, 13]. Less educated or lower-income families, who may benefit the most, could face access barriers due to limited smartphone proficiency, inconsistent connectivity, or competing caregiving demands [15].

While 89.7% of respondents expressed trust in digital data management, a notable minority remained uncertain. This aligns with previous literature emphasizing that trust, privacy, and perceived security are decisive predictors of sustained user engagement [14]. Building confidence requires robust data-protection frameworks, transparency in data use, and culturally sensitive education addressing privacy misconceptions [16].

Another recurrent theme involved requests for additional functionalities such as prescription refills or appointment scheduling. While these suggestions illustrate high digital expectations, they also highlight a boundary issue: integration must be carefully managed to ensure that DPEMS complement, rather than duplicate, existing hospital information systems. Seamless interoperability with institutional electronic health records is essential for sustainability and clinical credibility [15].

Future use and changing the face of medical management

The evidence from this study positions DPEMS as a pivotal innovation in the transformation of chronic-disease management, particularly in pediatric SCA. By providing continuous connectivity between families and healthcare teams, such platforms operationalize the World Health Organization’s vision for “connected care” and digital inclusivity [6]. Future versions could incorporate artificial intelligence-driven analytics to interpret patient-reported data, identify early warning signals (e.g., pain escalation, fever onset), and trigger preemptive clinical actions before crises occur [17].

In addition, the aggregation of longitudinal adherence and symptom data can inform precision public health, enabling stratified risk monitoring and resource allocation across SCA populations. Over time, digital platforms are likely to redefine the physician-patient interface - from episodic, encounter-based visits toward continuous, proactive, and participatory care [10, 17]. This transition represents not merely a technological evolution but a paradigm shift in the culture of pediatric chronic disease management, emphasizing empowerment, transparency, and shared accountability.

Limitations

This study has several limitations. It was conducted in a single tertiary center with a predominantly well-educated caregiver population, which may limit generalizability to settings with lower literacy or digital access. As a cross-sectional survey, it captures perceptions and readiness at one time point without assessing actual behavioral change or post-implementation outcomes. Self-reported adherence data are subject to recall and social-desirability bias. Finally, while qualitative insights were rich, they were limited to open-ended questionnaire responses rather than in-depth interviews.

## Conclusions

This study demonstrates high caregiver readiness for digital health adoption in pediatric sickle cell anemia care at the Armed Forces Hospital Southern Region, with strong agreement on usefulness, trust, and willingness to adopt, supporting both feasibility and demand. Digital systems offer clear potential to improve adherence, communication, and continuity of care, provided that barriers related to access, equity, and integration are addressed.

Future work should focus on implementation and outcome-based evaluation of clinical and quality-of-life measures, as digital health is likely to become a core component of comprehensive, patient-centered SCA management.
